# Prognostic role of long non-coding RNA LINC00152 in Chinese cancer patients: a meta-analysis

**DOI:** 10.18632/oncotarget.21838

**Published:** 2017-10-12

**Authors:** Liyang Liu, Jianfei Wen, Xi Gu, Dongdong Wu, Ming Lu, Qinghong Zhao

**Affiliations:** ^1^ Department of General Surgery, The Second Affiliated Hospital of Nanjing Medical University, Nanjing, Jiangsu, China; ^2^ Department of General Surgery, The First Affiliated Hospital of Nanjing Medical University, Nanjing, Jiangsu, China

**Keywords:** LINC00152, cancer, metastasis, prognosis, meta-analysis

## Abstract

The role of long intergenic non-coding RNA 152 (LINC00152) in predicting the prognosis of cancer has been investigated but results remain inconclusive and inconsistent. A meta-analysis was performed to explore the effect of LINC00152 on cancer prognosis. PubMed and ScienceDirect were searched for suitable studies and the results of 10 studies with a total of 775 patients were pooled. Pooled hazard ratios (HRs) and odds ratios (ORs) were calculated to assess the prognostic value of LINC00152. The results revealed that tumour patients with high LINC00152 expression were more likely to have lymph node metastasis (OR = 2.94, 95% CI 1.97–4.40, *P* < 0.001) and unfavourable tumour–node–metastasis stage (grade III/IV vs. I/II: OR = 3.07, 95% CI 1.69–5.59, *P* < 0.001). In addition, high LINC00152 expression levels were significantly associated with poor overall survival (pooled HR = 1.99, 95% CI 1.54–2.56, *P* < 0.001). The results suggest that high LINC00152 expression may serve as a predictive biomarker for the poor prognosis of various cancers in the Chinese population.

## INTRODUCTION

Although cancer mortality rates have continued to decline for several years, cancer is still a major cause of mortality worldwide [1, 2]. A total of 1 688 780 new cancer cases and 600 920 cancer deaths were projected to occur in the United States in 2017 [3]. In spite of major improvements in diagnosis and treatment, the 5-year survival rate remains low for many types of cancer [4]. Patients may have a better chance of recovering if cancer can be detected at an early stage, as appropriate treatment can be given in time. However, in patients with tumour metastasis at diagnosis, the effectiveness of therapy is limited. The identification of a new biomarker for predicting tumour metastasis and prognosis is, therefore, urgently required.

Long non-coding RNAs (lncRNAs) are generally defined as RNA transcripts that are longer than 200 nucleotides, and most lack protein coding capability [5]. LncRNAs have been found to alter gene expression through diverse molecular mechanisms [6], and participate in a variety of biological processes [7]. Although the function of lncRNAs is still being debated [8], they are considered a new class of regulatory non-coding RNAs. Accumulating evidence indicates that the dysregulation of lncRNAs plays a vital role in a large range of diseases, especially in cancers [9].

Long intergenic non-coding RNA (lincRNA) 152 (LINC00152), an 828 base pair lncRNA that locates to chromosome 2p11.2, was originally found hypomethylated in human hepatocellular carcinoma [10]. Since then, the up-regulation of LINC00152 has been reported in a variety of cancers, including lung adenocarcinoma [11], gastric cancer [12], renal cell carcinoma [13], gallbladder cancer [14] and tongue squamous cell carcinoma [15]. Up to now, numerous studies have demonstrated that the dysregulation of LINC00152 is correlated with clinicopathologic characteristics and patients' survival time in many types of cancer. However, most individual studies have been limited by small sample sizes and controversial results. To further assess the influence of LINC00152 on the clinical prognosis of multiple cancers, we conducted this meta-analysis.

## RESULTS

### Study characteristics

**Figure 1:**
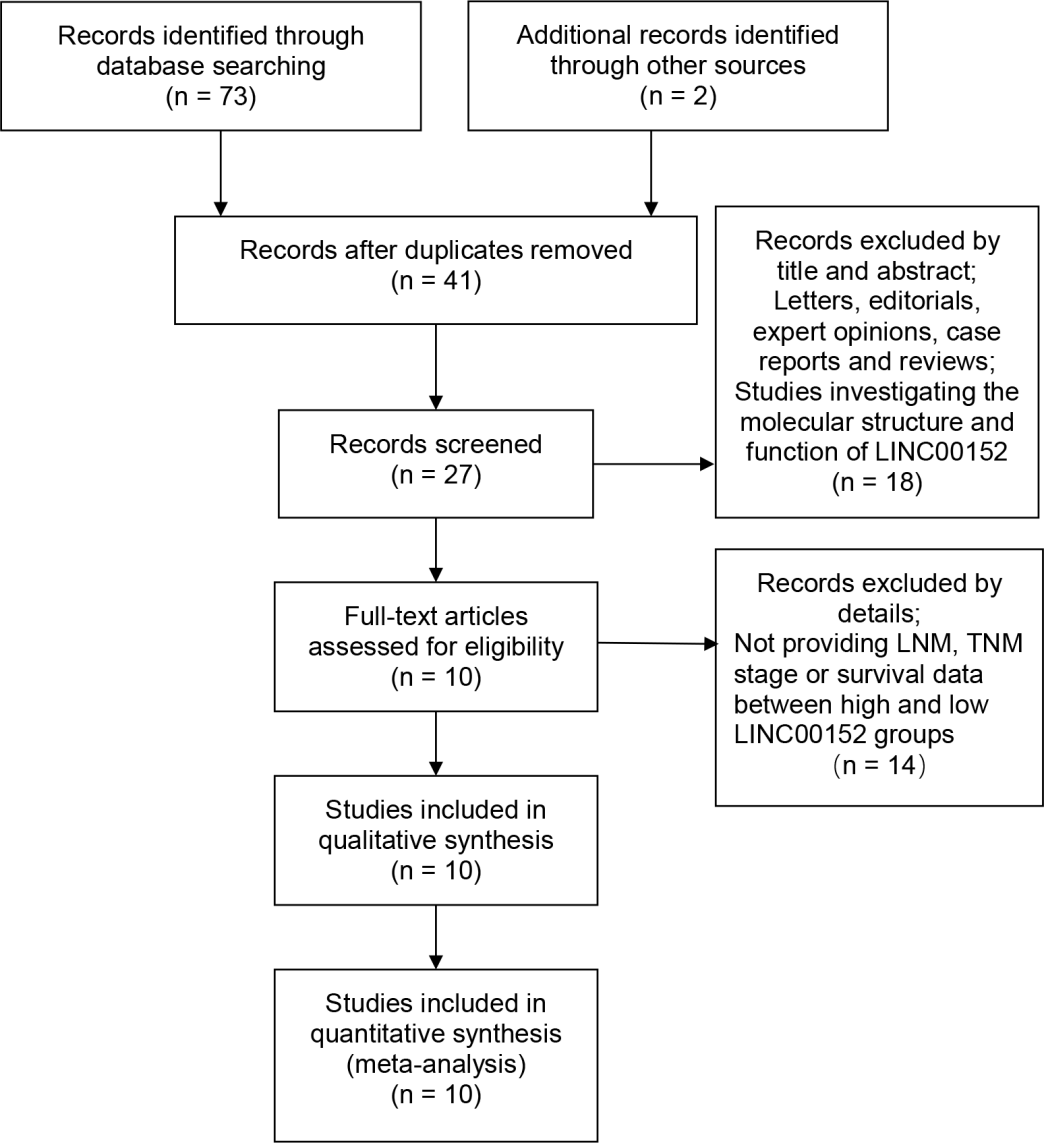
Flow diagram showing study selection

A total of 73 relevant publications were obtained from PubMed and ScienceDirect databases. Following application of the inclusion and exclusion criteria, 10 eligible articles were finally enrolled (Figure 1). The cancer types recorded in the included studies were tongue squamous cell carcinoma [15], lung cancer [16], lung adenocarcinoma [11], clear cell renal cell carcinoma [13], renal cell carcinoma [17], gallbladder cancer [14, 18], gastric cancer [12] and hepatocellular carcinoma [19, 20]. All the studies were from China. The characteristics of the eligible studies are shown in Table 1.

**Table 1:** Characteristics of included studies

Study	Year	Cancer type	LINC00152 expression						LINC00152 assay	HR statistic	HR (95% CI) high/low	NOS	Cut-off value	Follow-up (months)
			High			Low								
			Total	LNM	III/IV	Total	LNM	III/IV						
Feng [16]	2017	LC	51	-	-	50	-	-	qRT-PCR	Survival curve	2.04 (0.93–4.48)	8	ROC	60
Chen [11]	2017	LAD	30	25	22	30	5	8	qRT-PCR	Survival curve	2.16 (0.77–6.03)	8	Median ratio	40
Wu [13]	2016	ccRCC	38	-	-	39	-	-	qRT-PCR	Date in paper	2.58 (1.23–5.39)	7	-	> 60
Wang [17]	2017	RCC	27	19	21	18	7	6	qRT-PCR	Survival curve	1.98 (0.66–5.89)	8	Median ratio	> 60
Cai [14]	2016	GBC	23	15	17	17	5	6	qRT-PCR	-	-	8	Ratio of T/N (2)	-
Yu [15]	2017	TSCC	95	45	90	87	28	73	qRT-PCR	Survival curve	2.67 (1.38–5.33)	7	a	> 60
Chen [12]	2015	GC	59	43	28	48	34	16	qRT-PCR	Date in paper	1.66 (1.01–2.73)	8	ROC	> 60
Cai [18]	2016	GBC	18	6	9	17	5	13	qRT-PCR	Survival curve	2.53 (0.80–8.01)	7	Median ratio	40
Deng [19]	2017	HCC	38	-	18	34	-	9	qRT-PCR	Survival curve	1.63 (0.96–2.79)	7	-	> 60
Li [20]	2015	HCC	33	-	14	33	-	2	qRT-PCR	-	-	8	Median ratio	-

Note: The dashes represent no data.

^a^A semi-quantitative scoring criterion for *in situ* hybridization was used in Yu's study. The final scores were regarded as low expression (0–1) and high expression (2–3).

III/IV: TNM grade III/IV; LNM: lymph node metastasis; LC: lung cancer; LAD: lung adenocarcinoma; ccRCC: clear cell renal cell carcinoma; RCC: renal cell carcinoma; GBC: gallbladder cancer; TSCC: tongue squamous cell carcinoma; GC: gastric cancer; HCC: hepatocellular carcinoma.

### Association between LINC00152 expression and TNM stage (III/IV vs. I/II)

**Figure 2:**
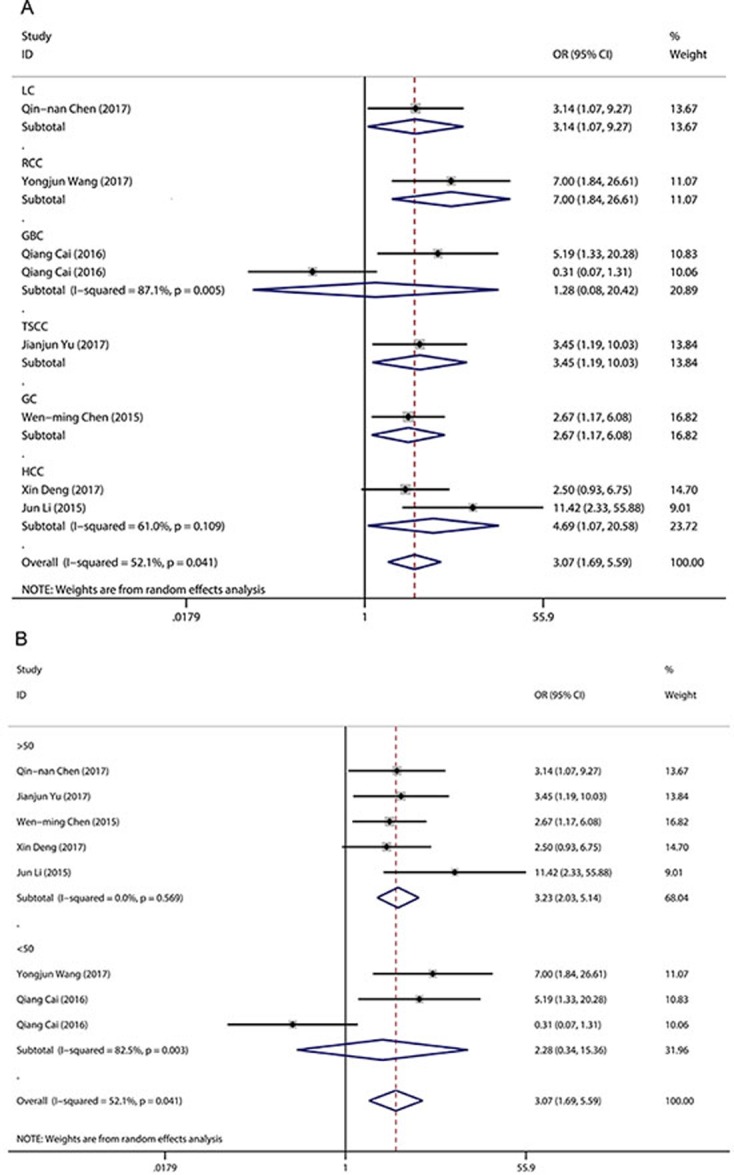
Forest plot for the association between LINC00152 expression and TNM stage (**A**) subgroup analysis of OR by cancer type; (**B**) subgroup analysis of OR by sample size.

A random-effects model was employed to analyse the pooled odds ratio (OR) and its 95% CI because of the existence of significant heterogeneity among those eight studies (*I*^2^ = 52.1%, *P* = 0.04) (Figure 2A). As a result, we found that elevated LINC00152 expression was predictive of unfavourable tumour–node–metastasis (TNM) stage (OR = 3.07, 95% CI: 1.69–5.59, *P* < 0.001). Due to the presence of heterogeneity, we performed stratified analyses of the data by cancer type and sample size. The results suggested that elevated LINC00152 levels were associated with higher TNM stage in hepatocellular carcinoma (OR = 4.69, 95% CI: 1.07–20.58, *P* =0.04) but not in gallbladder cancer (OR = 1.28, 95% CI: 0.08–20.42, *P* = 0.862). Furthermore, a significant association between TNM stage and LINC00152 expression was observed in a subgroup with sample sizes > 50 (OR = 3.23, 95% CI: 2.03–5.14, *P* < 0.001) (Figure 2B). The sample size of the included studies ranged from 35 to 187. When we chose 75 and 100 as the classification criteria for sample size, significant heterogeneity was observed in the subgroup with smaller sample sizes (data not shown).

### Association between LINC00152 expression and lymph node metastasis

**Figure 3:**
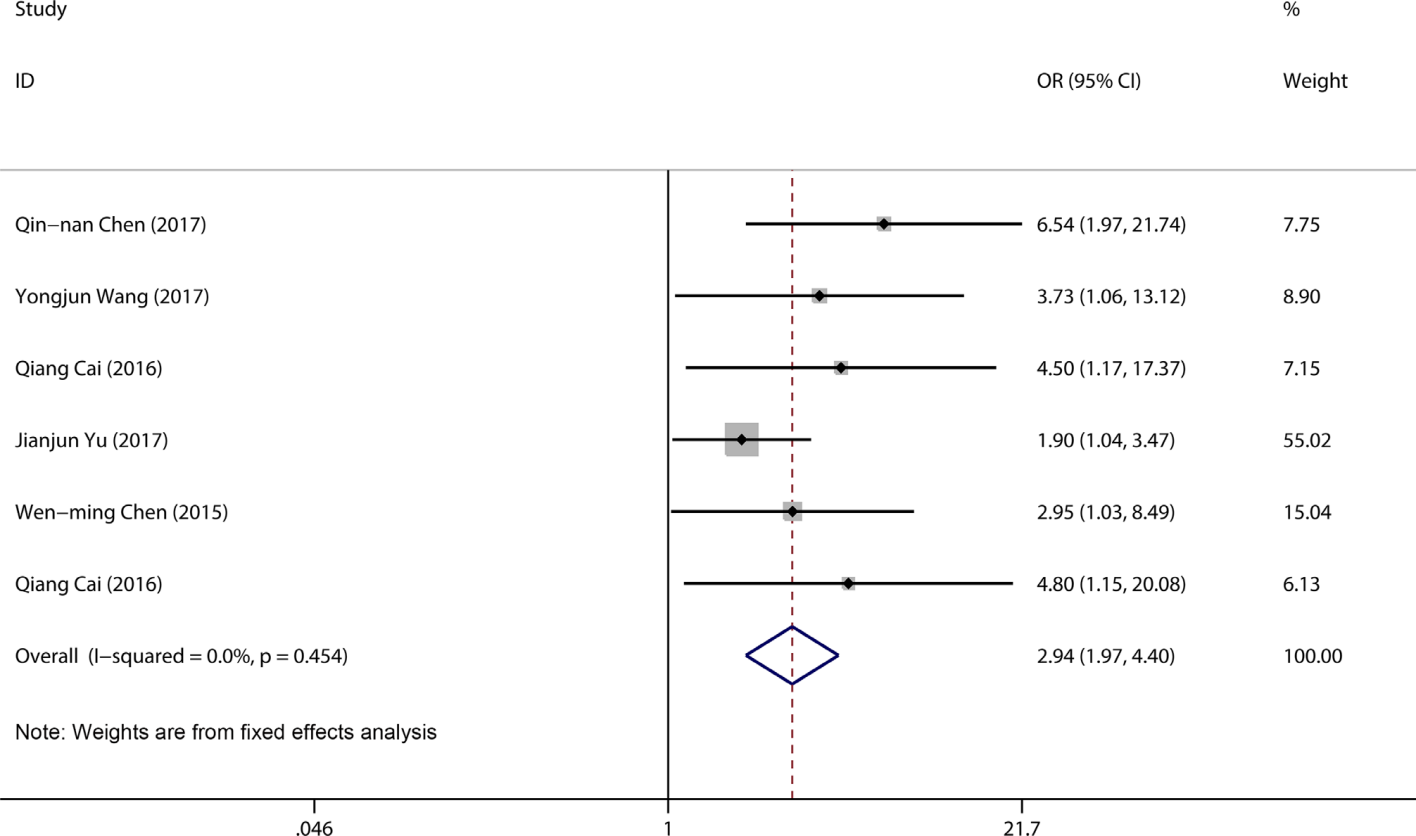
Forest plot for the association between LINC00152 expression and LNM

A total of 434 patients from six studies were pooled in a lymph node metastasis (LNM) group. We compared high LINC00152 expression with low LINC00152 expression using a fixed-effects model (*I*^2^ = 0.0%, *P* = 0.45). The pooled result revealed that patients with high LINC00152 expression were more likely to have lymph node metastasis (OR = 2.94, 95% CI 1.97–4.40, *P* < 0.001) (Figure 3).

### Association between LINC00152 expression and overall survival

**Figure 4:**
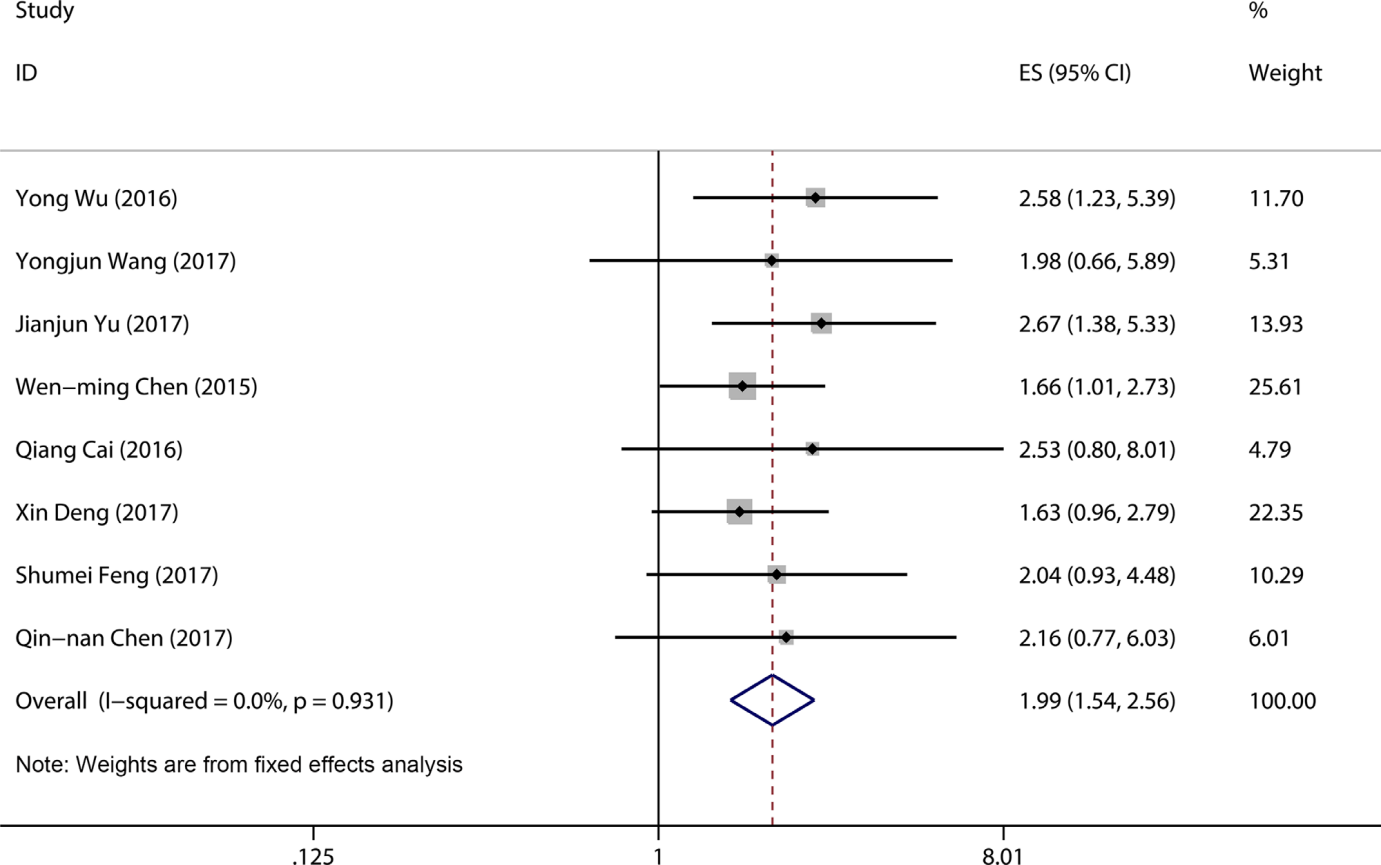
Forest plot of pooled HRs of elevated LINC00152 expression for OS

A total of six studies were used to obtain a pooled HR and 95% CI for overall survival (OS) in different cancer types. As no heterogeneity was detected in the OS group (*I*^2^ = 0.0%, *P* = 0.94), a fixed model was used. In this analysis, significant associations were observed between high LINC00152 expression level and poor OS (pooled HR = 1.99, 95% CI 1.54–2.56, *P* < 0.001) (Figure 4).

### Sensitivity analysis and publication bias

**Figure 5:**
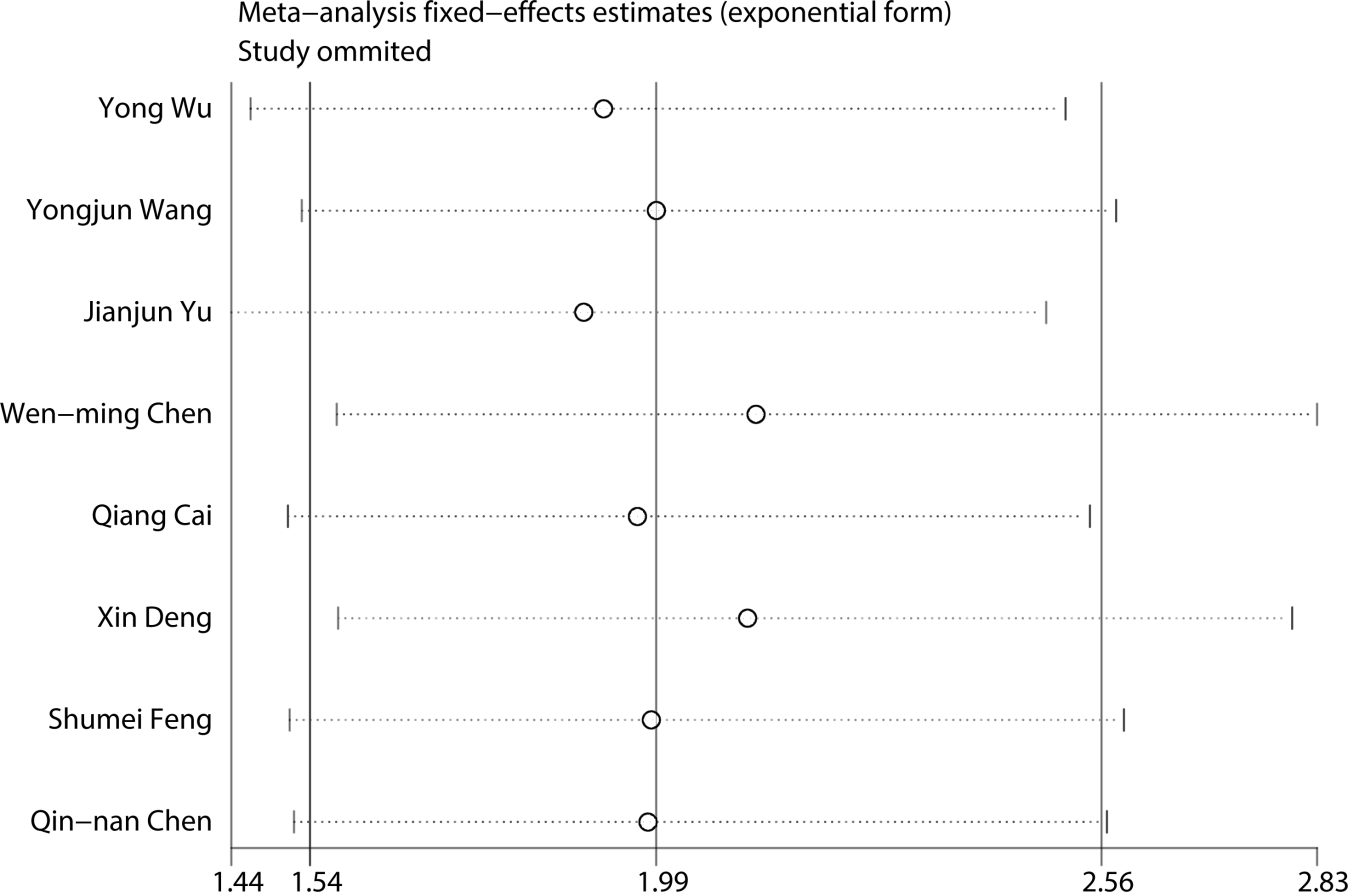
Result of sensitivity analysis

**Figure 6:**
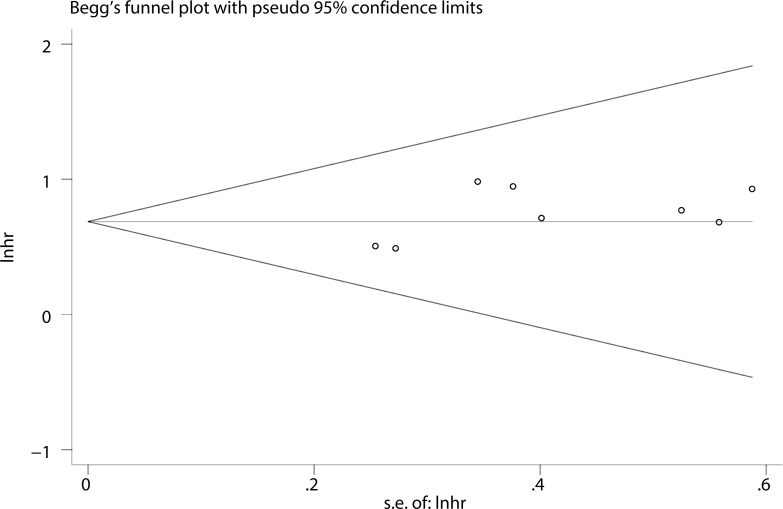
Funnel plot analysis of potential publication bias (Begg's test)

To evaluate the stability and credibility of our meta-analysis, a sensitivity analysis was performed on the OS group by omitting each study from analysis one at a time. The results indicated that no individual study significantly affected the pooled HR (Figure 5). In addition, the sensitivity analysis indicated that the pooled OR for elevated LINC00152 associated with TNM grade or LNM was not significantly affected by the exclusion of any of the studies (data not shown). Publication bias for the meta-analysis was evaluated using Begg's funnel plot analysis (*P* = 0.711) and Egger's linear regression test was performed to verify the accuracy of the Begg's funnel plot (*P* = 0.132). No obvious publication bias was detected by either test (Figure 6).

## DISCUSSION

LncRNAs are increasingly considered crucial players in a diverse range of human cancers, with large numbers of LncRNAs found participating in cancer-related biological processes [21–23]. It has been observed that the dysregulation of several lncRNAs is associated with the clinical outcome of a wide range of cancers; these lncRNAs include HOTAIR (Homeobox Transcript AntIsense RNA) [24], H19 [25], MALAT1 (Metastasis-Associated Lung Adenocarcinoma Transcript 1) [26], and UCA1 (Urothelial Carcinoma Associated 1) [27]. This suggests some lncRNAs have potential use as tumour biomarkers, forecasting prognosis and therapeutic efficacy.

LINC00152 is a newly identified lncRNA, and is currently a hot research topic. The mechanism by which LINC00152 promotes tumour progression is complex and remains to be further explored. Chen et al. [12] found that LINC00152 accelerated the cell cycle by binding to enhancer of zeste homologue 2 (EZH2) and silenced the expression of p15 and p21 in gastric cancer. Ji et al. [28] reported that LINC00152 promotes proliferation by targeting EpCAM via the mTOR signalling pathway. In addition, LINC00152 was found participating in some lincRNA–miRNA interactions. Wang et al. [17] found that LINC00152 negatively regulated miR-205 and promoted renal cell carcinoma progression. Cai et al. [18] demonstrated that LINC00152 could directly bind to miR-138, thus positively regulating HIF-1α, a target gene of miR-138, inducing cell metastasis and epithelial–mesenchymal transition in cancer cells.

A recent series of studies has explored the association between LINC00152 expression and the prognosis of various cancers, yielding inconsistent results. Considering the potential value of LINC00152, we conducted this meta-analysis of 775 patients from 10 studies to furtherly evaluate the prognostic utility of LINC00152 expression. The pooled results revealed that for tumour patients, higher LINC00152 expression was significantly associated with unfavourable LNM and TNM stage. By combining HRs from involved studies, we found that higher LINC00152 expression was indicative of poor OS time. Patients with high levels of LINC00152 expression had remarkably shorter survival time compared with those with lower levels. However, insufficient literature was available to enable us to perform a meta-analysis to examine whether higher LINC00152 expression in tumour issues may be related to disease-free survival, progression-free survival and event-free survival. Significant heterogeneity was observed in the TNM stage group. To clarify the source of heterogeneity, we conducted a subgroup analysis by sample size and cancer type. No significant heterogeneity was found in the subgroup with sample size > 50, and when the Cai [18] study was omitted, the only study with a sample size < 40, no significant heterogeneity was observed. We considered therefore that the difference in sample size may be the major source of heterogeneity; however, due to the small sample size of our meta-analysis, the exact source of heterogeneity could not be explained. In summary, our results suggest that LINC00152 could play an important role in cancer progression and could be used as a common biomarker for predicting clinical outcomes in cancer patients.

To the best of our knowledge, this is the first meta-analysis summarizing the relationship between LINC00152 levels and the prognosis of various cancers. However, the analysis was subject to limitations. Firstly, only 10 publications were included, making analysis less reliable than if a large sample had been available. Secondly, due to the limited literature, and unavailability of data in some of the included studies, we did not perform a subgroup analysis based on cancer type, age, sex or other factors. Thirdly, the included studies were based on the Chinese population. Therefore, our results may only account for this patient group and may not be applicable to other population groups. Fourthly, when HRs could not be obtained directly from the primary studies, estimated HRs extracted from the survival curves or calculated from the reported data were used for the pooled analysis, which weakened the reliability of our results. Finally, among the eligible studies, the cut-off value for high or low LINC00152 expression differed and this would need resolving before the clinical application of LINC00152 level.

In conclusion, the current meta-analysis revealed that elevated LINC00152 expression is significantly associated with LNM, unfavourable TNM grade and poor OS, in different cancer types. LINC00152 may serve as a common biomarker for predicting poor prognosis in patients with various cancers in the Chinese population.

## MATERIALS AND METHODS

### Publication search

We executed a comprehensive search of PubMed and ScienceDirect for relevant studies dating to 22 June 2017. The following keywords and terms were used for the search: “LINC00152” or “long intergenic non-coding RNA 152” and we searched only for studies published in full article form in English. The references of primary publications were also viewed to obtain additional relevant articles.

### Inclusion and exclusion criteria

Studies were included if they met the following criteria: (1) analysis of the prognostic role of LINC00152 in cancer patients was performed; (2) patients were grouped according to expression levels of LINC00152; (3) a link between LINC00152 expression and clinicopathologic parameters was included; and (4) sufficient data were provided to estimate HRs with a corresponding 95% CI for OS. Studies not fulfilling the criteria, as well as reviews and case reports, were excluded. Furthermore, if the same cohort was published more than once, only the most recent publication was included.

### Data extraction and quality assessment

Two authors independently reviewed each potentially relevant study and extracted data according to the inclusion and exclusion criteria. The following information was collected from the eligible studies: surname of first author; year of publication; cancer type; country; detection method; total number of patients; number of patients with LNM; number of high and low LINC00152 expression groups with different TNM stages; follow-up period; and HRs and corresponding 95% CIs for OS. Multivariate analysis effects were used when univariate and multivariate analysis were both provided. Disagreements were resolved through discussion among the authors. The Newcastle–Ottawa Scale (NOS) was conducted to assess the quality of the included studies, with an NOS score ≥ 6 considered high quality.

### Statistical methods

We evaluated the strength of the correlation between LINC00152 expression and clinical prognosis by pooled ORs or HRs with 95% CIs. Heterogeneity among studies was assessed by *I*^2^-based *Q* test and *I*^2^ index. We used a fixed-effects model if the *Q* test indicated an absence of prominent heterogeneity among the qualifying studies, defined as P_heterogeneity_ > 0.05 and/or *I*^2^ < 50% [29]. Otherwise, a random-effects model was used [30]. To estimate the stability of our results, sensitivity analyses were performed to assess the contributions of individual studies to the pooled HR. HRs and 95% CIs for OS were obtained from a Kaplan–Meier curve, using Engauge Digitizer 4.1 software [31]. Publication bias was evaluated by Begg's funnel plot, with *P* < 0.05 representing significant publication bias [32]. The statistical analyses were carried out with Stata software (version 12.0, Stata Corp, College Station, USA).
